# Preferred musical attribute dimensions underlie individual differences in music-induced analgesia

**DOI:** 10.1038/s41598-021-87943-z

**Published:** 2021-04-21

**Authors:** Krzysztof Basiński, Agata Zdun-Ryżewska, David M. Greenberg, Mikołaj Majkowicz

**Affiliations:** 1 Department of Quality of Life Research, Medical University of Gdańsk, Gdańsk, Poland; 2 Interdisciplinary Department of Social Sciences & Department of Music, Bar-Ilan University, Ramat Gan, Israel; 3 Autism Research Centre, Department of Psychiatry, University of Cambridge, Cambridge, UK; 4 Department of Health Science, Pomeranian University in Słupsk, Słupsk, Poland

**Keywords:** Psychology, Human behaviour

## Abstract

Music-induced analgesia (MIA) is a phenomenon that describes a situation in which listening to music influences pain perception. The heterogeneity of music used in MIA studies leads to a problem of a specific effect for an unspecified stimulus. To address this, we use a previously established model of musical preferences that categorizes the multidimensional sonic space of music into three basic dimensions: arousal, valence and depth. Participants entered an experimental pain stimulation while listening to compilations of short musical excerpts characteristic of each of the three attribute dimensions. The results showed an effect on the part of music attribute preferences on average pain, maximal pain, and pain tolerance after controlling for musical attributes and order effects. This suggests that individual preferences for music attributes play a significant role in MIA and that, in clinical contexts, music should not be chosen arbitrarily but according to individual preferences.

## Introduction

Pain is an unpleasant sensory and emotional experience associated with or resembling that associated with actual or potential tissue damage^1^. Although pain is often a consequence of the direct stimulation of nociceptors, this relationship is not always straightforward, and in the case of chronic pain, pain involves processes of neuroplasticity and cortical reorganization^2^. Many pain conditions have an unknown etiology and cognitive processes are implied in their persistence (e.g., lower back pain, irritable bowel syndrome, or fibromyalgia; see also the fear-avoidance model of pain)^3^. Nociception may be influenced by distraction or emotion via the descending pain modulatory systems (DPMS)^4^.

Music-induced analgesia (MIA) refers to the ability of music to alleviate pain, and it has been extensively studied in laboratory experiments^5–7^. A systematic review and meta-analysis of music interventions in postoperative care concluded that music can be administered to alleviate postoperative pain and reduce anxiety^8^. Similarly, a systematic review and meta-analysis of experimental studies on MIA indicated that music listening is effective for pain modulation^9^.

While the analgesic properties of music are well-studied, there is no consensus as to *what kind* of music (i.e., the specific musical attributes) is best for MIA. The music used in previous studies was either participant or experimenter chosen. In the first case, participants were asked to provide their own preferred music^6,7^, “pleasant and relaxing” favorite songs^10^, or “familiar, highly pleasant and slow paced” songs^11^. In the second case, a multitude of genres and labels were given for the music (i.e., easy listening, relaxing, soothing, classical, baroque, and sedative; see^8^ for a comprehensive list). One study^12^ used a quasi-selection method in which participants chose from a broad pool of pre-selected music but found no effect on the part of music attributes on pain outcomes. The heterogeneity of the musical stimuli used in MIA studies is a source of an important methodological problem, namely the assumption of “a specific effect for an unspecified stimulus”^13^.

The present study attempts to overcome this pivotal methodological constraint by leveraging a framework inspired by previous theory and research on musical preferences. Musical preferences can be conceptualized as a person’s affective or preferential response to a stimulus, typically measured with audio samples or genre-based labels. Previous research on musical preferences has typically been concerned with its underlying latent structure and external correlates^14,15^. Recent research has attempted to move beyond the concept of genres, which occupied the musical preference field in the 2000s^16^. Genre labels have methodological issues of their own, namely that they are arbitrarily defined labels determined by record companies and have social connotations. The most recent research has focused on conceptualizing and measuring musical preferences in terms of preferred music attributes^17^.

To better understand musical preferences in this way, researchers first measured the human perception of musical attributes. This research found that people’s perception of attributes spanning the multidimensional space of Western music can be organized into three basic factors: Arousal (the amount of energy perceived as being delivered by the music), Valence (the emotions, from negative to positive, perceived as being encouraged by the music), and Depth (the emotional and cerebral complexity perceived in the music)^17^. This model (the AVD model of musical attribute preferences) was shown to replicate within and across genres and to correlate with other conceptualizations of musical preferences^17^. It has also been replicated across geographic samples, stimuli sets, non-human computer-based extraction methods, and big data^18–20^. Preferences for these three musical attribute dimensions are correlated with personality traits and cognitive styles^17^. Taken together, the AVD model is a robust framework in which to study MIA.

Several mechanisms for MIA have been proposed: distraction, positive affect, relaxation, and reward system activation^5,9,10,21^. Music preferences may significantly influence all these mechanisms. Preferred music could be more engaging to the listener (providing more distraction), induce positive affect (irrespective of the music’s actual emotional tone), provide more relaxation, and be more rewarding. We suggest that these effects are mainly driven by preferences for various music attributes, not the attributes themselves. For example, a “sad” piece of music may be a source of distraction, relaxation, reward, or (paradoxically) positive emotions for someone if that person has a preference for negatively valenced music. Conversely, the same piece of music may not have such strong effects for a different person with a preference for positively valenced music. Thus, our aim in this study was to verify the hypothesis that music with preferred attributes produces a stronger analgesic effect than music with less-preferred attributes.

## Methods

### Participants

The participants in the study were 78 healthy volunteers. The participants were recruited from advertisements placed throughout campus and were mainly students and university staff. Two participants were excluded because they reported no pain during the procedure. Of the remaining 76, 47 were female (61.8%), and 29 were male (38.2%). The mean age of the participants was *M* = 28.16 (*SD* = 12.92). Forty-nine (64.4%) participants had completed secondary education (high school, technical school), while 27 (35.5%) had completed tertiary education. Before recruitment, participants were screened for the following exclusion criteria (Jackson et al. 2005): hearing deficits, diabetes, chronic pain conditions, circulatory disorders, hypertension, Raynaud’s disease, previous cold injury, blood clotting problems, and pregnancy. Professional musicians and participants with formal musical training of over six years (equivalent to primary-level music school in the Polish education system) were also excluded. The mean years of music education (either private tutorship or music school) was *M* = 1.25 (*SD* = 2.04).

### Musical stimuli

Each participant listened to three sets of musical stimuli, each comprised of eight 15-s excerpts taken from previous studies^17,22^. Each of the three sets was prepared in such a way to as reflect each of the three music attribute dimensions (Arousal, Valence, and Depth). The excerpts were chosen from a larger pool based on previously established factor loadings^17^. We chose songs that had a high factor loading (more than + 1 SD) for a target factor, while loading average (between − 1 and + 1 SD) on both other factors. We have thus obtained three relatively short sets of highly differentiated music excerpts that were characteristic to each of the three music attribute dimensions (see Appendix 1). To ensure novelty, music stimuli were comprised of either unreleased songs bought especially for a previous study^22^ or songs that were released commercially but had very low sales figures. After the experiment, participants were asked if they recognized any of the music. No participants indicated they were familiar with any of the presented music.

The order of the excerpts was randomized for each participant and condition. In the control condition, participants listened to white noise. All stimuli were normalized for loudness using ReplayGain^23^ and played back at a comfortable participant-chosen volume (the volume was set using unrelated music with the peak loudness normalized to the experimental stimuli). All sound stimuli were delivered using a laptop PC with an external audio interface (Focusrite Scarlett Solo, Focusrite plc) and a pair of studio-grade over-ear headphones (AKG K612 Pro, AKG Acoustics).

### Pain stimuli

Pain stimulation was performed with a cold-pressor test, a widely used, safe, and reliable method of experimental pain induction used in previous studies on MIA^7,21^. Subjects submerged their non-dominant hand up to their wrists in cold water and were asked to keep it there until they were too uncomfortable to continue. For safety, maximum stimulation time was set to 120 s. Subjects were asked to provide a verbal indication when they started to feel pain. A circulatory water bath (Jeiotech Inc.) was used to provide constant temperature throughout the study. Circulation was set to the maximum to avoid local water heating in the vicinity of the hand. Water temperature was set to 3 °C based on the results of Mitchell et al.^24^ to provide an average stimulation time of around 60 s.

### Measures

Music preference was rated on an 11-point Likert scale response to the question “How much did you like the music that you have just heard?” Pain threshold was measured as the time elapsed until the subject indicated pain. Pain tolerance was measured as the overall trial length (until the subject was too uncomfortable to continue). After each stimulation, participants rated the intensity of their pain using an 11-point numerical rating scale from 0 (*no pain*) to 10 (*worst possible pain*). These ratings were given for maximal pain (“when it was the worst”), average pain (“on average during this trial”), and the controllability of pain (from 0—*I had it totally under control* to 10 – *it was totally uncontrollable*). Physiological measures of arousal (blood pressure and heart rate) were taken before the procedure and after each trial.

### Procedure

The procedure followed the guidelines of the Declaration of Helsinki and was approved by the Independent Bioethics Commission for Research of the Medical University of Gdańsk, Poland. Participants were recruited from adverts on University campus, mailing lists, and local news websites. They were instructed during recruitment not to take any pain medication for the 24 h prior to the study. Upon entering the lab, the participants were briefed on the procedure and the data to be gathered and given an informed consent form.

The study utilized a within-subjects design. Each participant completed three music trials (arousal, valence, and depth) and a control trial (white noise). For each subject, the order of the trials and the order of songs within a trial was randomized. The music was played concurrently with the cold-pressor test. The music stopped as soon as the participant removed his or her hand from the water bath. To minimize the effects of cold on consecutive trials, there was a rest period after each of the trials. The length of the rest period was set at 5 min, after which the participants were asked whether they had stopped feeling pain and were ready to continue. The rest period was extended as needed. During the rest period, participants submerged their hands in warm water (34–36 °C) and completed the pain and music preference measures, and physiological measurements were taken.

### Statistical analyses

Data analysis was performed in Python with the following packages: NumPy and Pandas for data processing^25,26^, Statsmodels for statistical analysis^27^, and Seaborn for plotting^28^. A mixed-models analysis was performed in R^29^ using the package *lme4*^30^. The p-values for the models were calculated using the package *lmerTest*^31^. Monte Carlo simulations for power analysis were made using the package *simr*^32^. For each participant and each pain outcome measure (max pain, average pain, controllability, tolerance, and threshold), the score in the control condition was subtracted from the music condition scores. The resulting delta score is positive if the score increased in comparison with the control. Similarly, the delta is negative if the score decreased. For each pain outcome, a mixed-effects linear regression analysis was performed with the delta pain score as a dependent variable and music preference as a fixed effect. The participant was entered as a random effect to account for individual differences in pain perception. To control for trial order effects and the variability resulting from a given music condition, these were also inserted into the models as fixed effects. For music condition (a categorical variable), arousal was set as a reference. Overall, the models were constructed from a general pattern:

$$Outcome \sim music\;preference + music\;condition + trial\;order + \left( {1|subject} \right)$$

where *outcome* is average pain, maximal pain, pain controllability, pain threshold, or pain tolerance. *1|x* denotes a random effect.

The power analysis indicated that a sample size of N = 31 would be sufficient to observe an effect of a two-second increase in pain tolerance resulting from a one-point increase in music preference in 80% of cases (Monte Carlo simulation with 1000 iterations, effect size: $\beta$ = 2; p < 0.05). A larger sample size was deemed necessary in order to capture enough variance in music attribute preferences.

## Results

Mean average pain, maximal pain, and pain controllability scores were lower in all of music conditions than in the control condition. Most average pain scores fell near the middle of the 11-point NRS scale (*M* = 5.11, *SD* = 1.96 for Arousal; *M* = 4.96, *SD* = 1.88 for Valence; *M* = 4.92, *SD* = 1.86 for Depth; *M* = 5.51, *SD* = 2.12 for control). Threshold and tolerance times were higher in all of the music conditions than in the control condition. Mean tolerance times ranged from 52.21 (*SD* = 41.14) in the control condition to 61.67 (*SD* = 41.45) in the Depth condition. Tolerance times showed substantial variance and, in five trials, reached the maximum limit of 120 s. The means and standard deviations of pain outcome scores are presented in Table 1.

**Table 1:** Means and standard deviations of pain scores.

Pain	Arousal	Valence	Depth	Control
Average	5.11 (1.96)	4.96 (1.88)	4.92 (1.86)	5.51 (2.12)
Maximal	6.72 (2.08)	6.54 (2.04)	6.72 (2.11)	6.99 (2.02)
Controllability	4.33 (2.57)	4.47 (2.52)	4.55 (2.45)	4.68 (2.56)
Threshold	21.3 (23.32)	20.45 (22.22)	20.51 (21.73)	17.5 (18.69)
Tolerance	61.28 (41.43)	58.88 (41.48)	61.67 (41.45)	52.21 (41.14)

**Figure 1:**
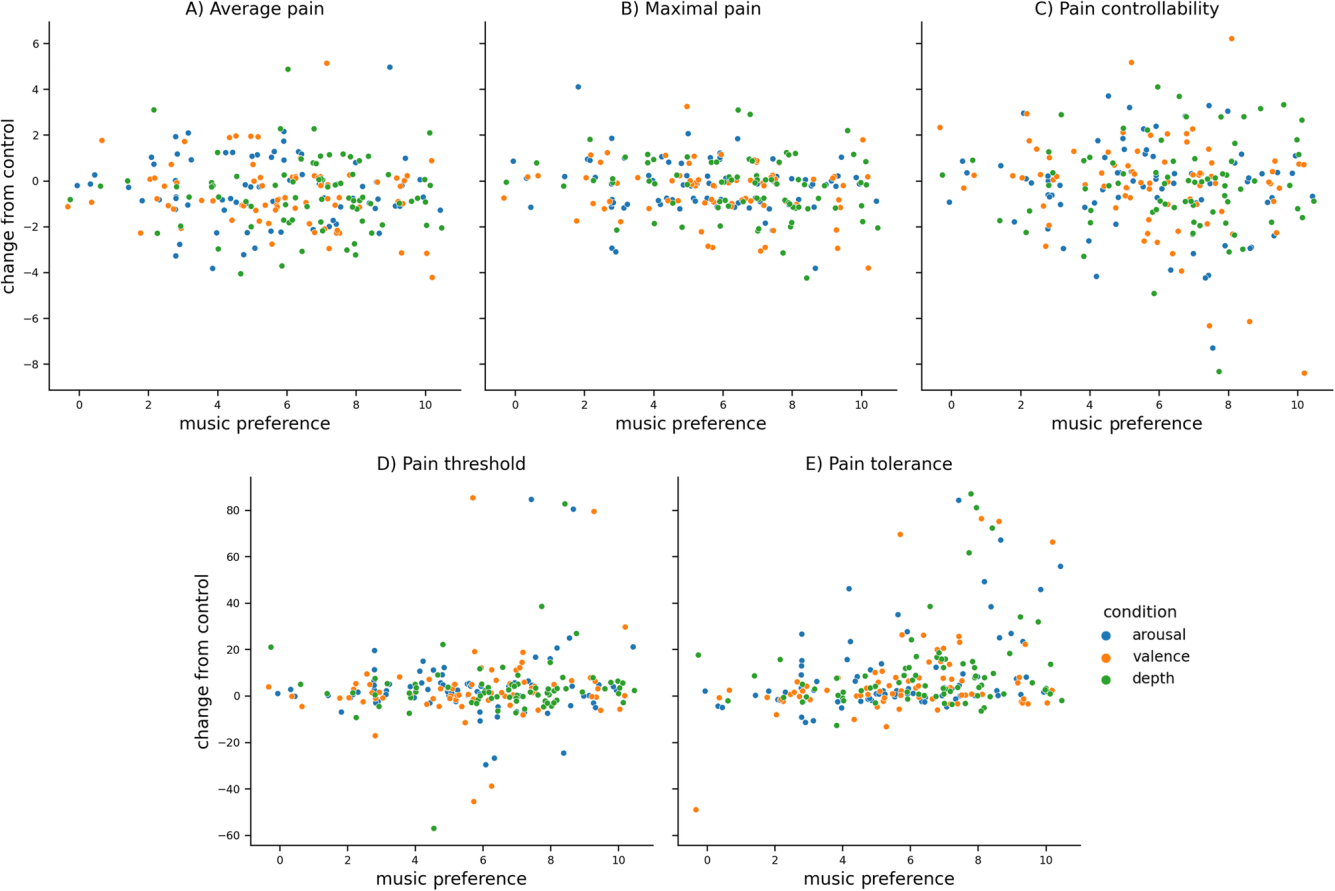
Pain outcomes as a function of music preference. Each data point represents one trial by one participant. Outcomes are reported as differences between one of three music conditions and the control condition. A value of 0 indicates no change from control. Random jitter was applied to all data points to facilitate the visualization of overlapping points. Presented outcomes are (**a**) average pain, (**b**) maximal pain, (**c**) pain controllability, (**d**) pain threshold, and (**e**) pain tolerance.

Mixed-effects regression (Fig. 1, Table 2) revealed statistically significant effects on the part of music preference on pain in three out of five studied pain measures. Preferred music was associated with lower average pain (Fig. 1a, $\beta$ = − 0.31, *SE* = 0.03, *p* = 0.002), lower maximal pain (Fig. 1b, $\beta$ = − 0.09, *SE* = 0.03, *p* = 0.001), and higher pain tolerance (Fig. 1d, $\beta$ = 1.93, *SE* = 0.44, *p* = 0.0001). These effects were present after controlling for music condition and order of presentation. Pain controllability was not significantly predicted by music preference (Fig. 1c, $\beta$ = − 0.06, *SE* = 0.05, *p* = 0.193). Pain threshold was also not predicted by music preference (Fig. 1e, $\beta$ = 0.41, *SE* = 0.36, *p* = 0.257).

**Table 2:** Linear mixed effects regression analyses.

Outcome	Predictor	$\beta$	SE	p
Average pain	Intercept	− 0.31	0.25	0.222
	Music preference	− 0.10	0.03	0.002**
	Condition (depth)	− 0.12	0.14	0.402
	Condition (valence)	− 0.13	0.14	0.365
	Order	0.19	0.05	0.001***
Maximal pain	Intercept	− 0.06	0.21	0.775
	Music preference	− 0.09	0.03	0.001***
	Condition (depth)	0.07	0.12	0.585
	Condition (valence)	− 0.16	0.12	0.177
	Order	0.13	0.05	0.009**
Controllability	Intercept	0.07	0.37	0.844
	Music preference	− 0.06	0.05	0.193
	Condition (depth)	0.29	0.22	0.176
	Condition (valence)	0.17	0.21	0.433
	Order	− 0.04	0.08	0.627
Pain tolerance	Intercept	− 6.60	3.36	0.051
	Music preference	1.93	0.44	0.0001 ***
	Condition (depth)	− 1.98	2.10	0.348
	Condition (valence)	− 3.12	2.06	0.132
	Order	2.13	0.79	0.008**
Pain threshold	Intercept	− 1.85	2.85	0.516
	Music preference	0.41	0.36	0.257
	Condition (depth)	− 1.49	1.70	0.384
	Condition (valence)	− 1.07	1.67	0.521
	Order	1.41	0.65	0.030*
Heart rate	Intercept	− 0.38	1.95	0.845
	Music preference	− 0.31	0.23	0.177
	Condition (depth)	− 1.33	1.05	0.205
	Condition (valence)	0.08	1.02	0.942
	Order	− 1.30	0.40	0.001***
Blood pressure (systolic)	Intercept	− 7.20	2.91	0.014*
	Music preference	0.17	0.37	0.648
	Condition (depth)	2.92	1.69	0.086
	Condition (valence)	1.64	1.65	0.324
	Order	− 1.24	0.64	0.055
Blood pressure (diastolic)	Intercept	− 2.34	1.29	0.072
	Music preference	− 0.17	0.16	0.274
	Condition (depth)	1.37	0.71	0.055
	Condition (valence)	1.40	0.69	0.045*
	Order	0.05	0.27	0.849

Music preference, music condition, and experimental order as predictors of five pain outcomes. *Beta* regression coefficient; *SE* standard error. Intercept values are for the arousal condition.

* p < 0.05; ** p < 0.01; *** p < 0.001.

Similar mixed-effects regression models were built for heart rate and systolic and diastolic blood pressure (Table 2). No significant effects on the part of music preference were observed. Trial order was associated with heart rate (lower in later trials, $\beta$ = − 1.30, *SE* = 0.40, *p* < 0.001). Valence condition was associated with higher diastolic blood pressure ($\beta$ = 1.40, *SE* = 0.69, *p* < 0.05).

## Discussion

The results of this study point to a significant role of music attribute preferences in MIA. Specifically, we found that listening to music with preferred attributes reduced pain intensity (average as well as maximal) and increased pain tolerance. These results support our initial hypothesis. Importantly, the effects of music attribute preferences were present after controlling for the actual music condition. This result may be interpreted as evidence that what is crucial to the level of MIA is the individual’s affective response to the music and not the music itself. It is therefore entirely plausible that the same piece of music will produce a strong analgesic effect in one person and no effect whatsoever in another, depending on the individual differences in music attribute preferences.

While statistically significant, the observed effects may be considered small (in the case of average and maximal pain) to moderate (pain tolerance). This is expected because the overall effects of MIA are moderate in most studies (see^9^ for a meta-analysis). The effect size of pain tolerance could have also been limited due to ceiling effects related to the two-minute maximum stimulation time. This was the case in five trials, in which participants indicated that they were happy to continue for longer than two minutes and wanted to hear more music despite the pain.

Effects on pain controllability were not significant. This may be due to the concept of pain controllability being difficult to understand for the participants (some indicated during debriefing that they were not sure about the exact meaning of controllability). The related concept of pain unpleasantness may be a more suitable outcome measure because it may be easier to understand. Music has also been shown to influence pain unpleasantness more significantly than pain intensity^33^. Furthermore, pain thresholds were unaffected by music attribute preference. One important aspect of this work is that the music used to assess attribute preferences was novel to the participants. Autobiographical memories related to familiar songs may elicit powerful emotions^34^. Familiarity may also play a mediating role in MIA because predictions and violations of predictions about music are associated with reward circuit activation^35^. The methodology used in this study overcomes the problem of familiarity by using music that is novel while at the same time having an established attribute structure.

The effects of trial order were statistically significant for most outcome measures. This was expected because multiple consecutive cold pressor tests tend to influence pain outcomes^24^. In this study, consecutive cold pressor stimulations increased average and maximal pain scores, while at the same time (somewhat paradoxically) increasing pain tolerance and thresholds. To control for the bias that this may have introduced, order was entered into the regression models as a fixed effect.

Recent advances in digital technology bring musical libraries to people’s fingertips and present a new frontier in which music can be used in medical and therapeutic setting^36^. One practical application of our results may be in the development of new therapeutic tools for patients suffering from chronic pain conditions. In fact, MIA is especially valid in these cases because music is safe, cheap, and simple to use and could be administered for prolonged periods of time. One challenge involved in music-based interventions is avoiding the boredom caused by prolonged listening to the same pieces. Music streaming services already use listener profiling to provide recommendations. Using similar recommendation systems based on music attribute preferences in therapeutic settings may be greatly beneficial for those with chronic pain conditions. These types of recommendation systems can be the foundation for data-driven mobile applications that can supplement medical and therapeutic treatments between sessions with the professional practitioners.

Another future avenue for research is to understand the role of individual differences in how personality impacts MIA. Research building on interactionist theories^37^ has shown that individual differences in musical preferences regarding both broad styles and specific attributes are associated with personality traits and cognition^17,38–40^. Furthermore, recent research has shown evidence for the self-congruity effect of music, whereby listeners prefer the music of artists who have similar personal characteristics as themselves^41^. Future research should therefore explore the role of personality in MIA.

One potential limitation of this study is the use of only the “positive” side of music attribute dimensions (that is, only music that ranked highly on one of the attribute dimensions was used). This was done to limit the number of trials each participant was subjected to. The cold-pressor procedure is very unpleasant to some participants, and while no one aborted the procedure, some indicated that they would not complete more than four trials. It is possible that other combinations of music dimensions (for example, “high-arousal, low-depth”) might have produced specific interaction effects that were not investigated in this study. Future studies may consider applying a full 2 × 2 × 2 factorial design for each musical dimension, yet a less burdensome pain stimulation procedure would have to be employed (e.g., rapid heat stimuli using thermodes).

## Data Availability

The experiment was not formally preregistered. De-identified data for this study, along with a Jupyter Notebook with the data analysis scripts, can be accessed in an Open Science Framework repository: https://osf.io/pqrjs/?view_only=0448153a2ea5467680cd4da6bdbd710a.

## Appendix 1

**Table:**

Condition	Artist	Title	Filename
Arousal	Five Finger Death Punch	White Knuckles	147989_Getty_15.mp3
	Straight Outta Junior High	Over now	113830_Getty_15.mp3
	Ornette Coleman	Rock The Clock	47.mp3
	Laurent Martin	Scriabin Etude Opus	87292_Getty_15.mp3
	Bankrupt	Face the Failure	112632_Getty_15.mp3
	Elliot Carter	Boston Concerto: Allegro Staccatissimo	8.mp3
	Dawn Over Zero	Out of Lies	90349_Getty_15.mp3
	Five Finger Death Punch	Death Before Dishonor	147991_Getty_15.mp3
Depth	Farrenc	Piano quintet no 1 in a minor	903.mp3
	Philip Glass	Symphony No3	904.mp3
	Ruben Gonzalez	Zancudo	905.mp3
	William Boyce	Symphony #1	3.mp3
	Bruce Smith	Sonata A Major	110353_Getty_15.mp3
	Earl Klugh	Laughter in the rain	902.mp3
	Dna	La Wally	62187_Getty_15.mp3
	Mantovani	I Wish You Love	28.mp3
Valence	Brigitte	Heute Nact	5.mp3
	Herb Ellis And Joe Pass	Cherokee	9.mp3
	Curtis	Carrots and Grapes	58264_Getty_15.mp3
	Walter Legawiec And His Polka Kings	Bohemian beer party	907.mp3
	Doc Watson	Interstate Rag	26.mp3
	Hilton Ruiz	Mambo Numero Cinco	37.mp3
	Bill Haley And His Comets	Razzle Dazzle	46.mp3
	Flamin' Groovies	Gonna Rock Tonight	20.mp3

Audio files used in the study are included in the OSF repository (https://osf.io/pqrjs/?view_only=0448153a2ea5467680cd4da6bdbd710a).
